# Towards Elimination of Mother-to-Child Transmission of HIV: The Impact of a Rapid Results Initiative in Nyanza Province, Kenya

**DOI:** 10.1155/2012/602120

**Published:** 2012-04-04

**Authors:** Lisa L. Dillabaugh, Jayne Lewis Kulzer, Kevin Owuor, Valerie Ndege, Arbogast Oyanga, Evelyne Ngugi, Starley B. Shade, Elizabeth Bukusi, Craig R. Cohen

**Affiliations:** ^1^Department of Pediatrics, University of California San Francisco, San Francisco, CA, USA; ^2^Family AIDS Care and Education Services (FACES), Research, Care and Training Program, Kenya Medical Research Institute, Kisumu, Kenya; ^3^U.S. Centers for Disease Control and Prevention, Division of Global HIV/AIDS, Nairobi, Kenya; ^4^Obstetrics, Gynecology and Reproductive Sciences, University of California San Francisco, San Francisco, CA, USA

## Abstract

Many HIV-positive pregnant women and infants are still not receiving optimal services, preventing the goal of eliminating mother-to-child transmission (MTCT) and improving maternal child health overall. A Rapid Results Initiative (RRI) approach was utilized to address key challenges in delivery of prevention of MTCT (PMTCT) services including highly active antiretroviral therapy (HAART) uptake for women and infants. The RRI was conducted between April and June 2011 at 119 health facilities in five districts in Nyanza Province, Kenya. Aggregated site-level data were compared at baseline before the RRI (Oct 2010–Jan 2011), during the RRI, and post-RRI (Jul–Sep 2011) using pre-post cohort analysis. HAART uptake amongst all HIV-positive pregnant women increased by 40% (RR 1.4, 95% CI 1.2–1.7) and continued to improve post-RRI (RR 1.6, 95% CI 1.4–1.8). HAART uptake in HIV-positive infants remained stable (RR 1.1, 95% CI 0.9–1.4) during the RRI and improved by 30% (RR 1.3, 95% CI 1.0–1.6) post-RRI. Significant improvement in PMTCT services can be achieved through introduction of an RRI, which appears to lead to sustained benefits for pregnant HIV-infected women and their infants.

## 1. Introduction

Despite extensive scaleup of prevention of mother-to-child transmission (PMTCT) services in Sub-Saharan Africa, many HIV-infected pregnant women and their HIV-exposed infants are not receiving the complete package of preventive and treatment services they need to reduce the risk MTCT of HIV to <2% [1, 2]. Without effective PMTCT intervention, the risk of MTCT during pregnancy and birth is 15–50% and another 5–20% will become infected through breastfeeding. Estimates in 2009 revealed that among 1.4 million HIV-positive pregnant women, (over 90% of which were in Sub-Saharan Africa) only 53% had received antiretrovirals (ARVs) to reduce MTCT risk [3]. A substantial portion of HIV-positive pregnant women qualify for more than preventive ARVs and need highly active antiretroviral therapy (HAART) for their own health and to significantly reduce the risk of MTCT [3–5]. However, according to the World Health Organization (WHO), in 2009 only 51% of HIV-positive pregnant women had been clinically or immunologically assessed and among those eligible, only 15% were provided HAART [3, 4]. HIV-exposed children face a similar situation. Early infant diagnosis (EID) using DNA polymerase chain reaction (PCR) testing to determine infant HIV status is a matter of urgency in infancy, yet only 15% of exposed infants were tested within the first two months of life [3].

Successful delivery of PMTCT relies on a cascade of successful steps including HIV counseling and testing, assessment of HAART eligibility through CD4+ testing and clinical staging, and ARV prophylaxis (including HAART) provision, infant testing, and HAART provision for HIV-positive infants [5–9]. Considerable challenges interfere with PMTCT delivery, particularly with respect to HAART uptake for qualifying women and infants. These include health systems infrastructure limitations such as inefficient laboratory flow, inconsistent commodity supply for essential lab services or drugs, and poor provider knowledge [9–13]. Low stakeholder involvement, poor integration of services, and ineffective referrals are other important factors. Low patient, partner, and community PMTCT knowledge, as well as social stigma, fear, and denial, can also prevent women from accessing or following through with the HIV care and treatment they need [14, 15]. Collectively these challenges make provision of PMTCT services at each step difficult, contributing to compromised PMTCT quality of care.

Many strategies for improving health system delivery have been utilized in developing country settings such as continuous quality improvement (CQI) and Plan, Do, See, Act (PDSA). The Rapid Response Initiative (RRI) is an approach that has been refined over the past 40 years to affect organization change and improve performance. It is widely used by the World Bank to facilitate projects and optimize limited resources [16, 17]. The RRI approach borrows key components from the CQI and PDSA processes but focuses on achieving ambitious change rapidly with dramatic and sustainable results. An RRI includes a results-oriented approach, that is fast, multidisciplinary, empowering and fosters innovation and learning. RRI often include 2 key phases: (1) needs assessment and (2) implementation and monitoring. By structuring goals into short cycles (such as 60 days), programs can quickly introduce health system strengthening and improvement along with self-monitoring and real-time response to challenge areas. The limited time cycle allows programs to quickly review effective strategies and avoids workforce fatigue that may be associated with CQI or PDSA approaches. Many African communities have adopted the RRI approach with impressive results such as country-wide HIV program implementation in Eritrea and HIV testing and voluntary medical male circumcision in Kenya [18–20].

In order to address PMTCT service challenges, Family AIDS Care and Education Services (FACES) in collaboration with the Kenyan Ministry of Health (MOH) and other stakeholders developed and implemented an RRI to increase service provision and uptake, including HAART initiation for eligible HIV-positive mothers and infants. Key indicators were compared at baseline, during the RRI, and post-RRI to determine if this targeted approach resulted in improved PMTCT services.

## 2. Methods

### 2.1. Rapid Results Initiative Process

**Figure 1:**
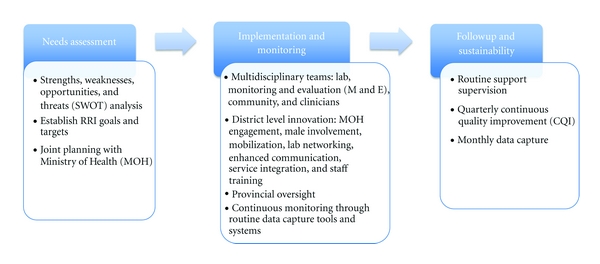
Rapid Results Initiative Approach.

 The RRI was structured in 3 stages: (1) needs assessment, (2) implementation and monitoring, and (3) followup for sustainability (Figure 1). First, a joint-needs assessment at a provincial level using a Strengths, Weaknesses, Opportunities, and Threats (SWOT) analysis was conducted by FACES and the Kenyan MOH in January 2011. Specific objectives were agreed on and targets set for each measurable outcome over a 60-day implementation timeframe scheduled to begin in April 2011. Specific objectives included (1) increase assessment of HAART eligibility and uptake amongst HIV-positive pregnant women, (2) improve uptake of testing for HIV-exposed infants, and (3) increase HAART uptake amongst HIV-positive infants.

The implementation phase was organized by provincial and district-level multidisciplinary taskforces including laboratory, monitoring and evaluation, community liaison and clinical staff. Strategies for achieving targets were formulated at both provincial and district levels and are described below. Followup and sustainability efforts are ongoing and include routine support supervision and monitoring of PMTCT activities conducted jointly by FACES and the MOH as well as continuous quality improvement exercises conducted quarterly.

### 2.2. Strategies

 In addition to strategic approaches for each objective, three overarching strategies were identified as cross-cutting to accomplish all three objectives: (1) MOH leadership and involvement through joint planning, implementation, and support supervision, (2) increased male partner involvement through invitational letters requesting male partners to accompany their pregnant partner to the clinic for HIV couples counseling and testing, and (3) focused community mobilization which involved engaging local opinion leaders, community health workers, and mass media on high impact days such as market days.

### 2.3. Objective 1 Strategies

 To increase HAART eligibility assessment among HIV-positive pregnant women, laboratory network for CD4 sample and result transport was harmonized between facilities and hubs; sample transport was increased to daily or twice weekly; access to cell stabilizer tubes was increased to allow for daily blood drawing at peripheral sites; and CD4 samples from ANC were flagged at the lab for prioritization. To increase HAART uptake for eligible women, health care providers were notified of eligible CD4 counts; ART was integrated into ANC clinics to increase access at some sites previously without ART; and cell phones were used to contact eligible women.

### 2.4. Objective 2 Strategies

 To improve exposed infant testing uptake, the focus was on (1) improved identification of HIV-exposed infants by prioritizing infant exposure status assessment in the mother's ANC record (national mother-child booklet), offering to test women of unknown HIV status, and conducting same-day dried blood spot sampling for PCR testing for exposed infants, (2) decreased turnaround time for PCR results through laboratory strengthening mentioned above, facility-level problem-solving to reduce delays, and use of mobile phones to rapidly communicate positive results, and (3) staff training and mentorship was conducted.

### 2.5. Objective 3 Strategies

 To increase HAART uptake amongst HIV-positive infants. HIV-positive PCR results were flagged at district hospital labs and immediately communicated by phone to facilities. Facility staff then contacted parents by phone or sent a community health worker to notify parents of the HIV-positive results and ensure return to the facility. Staff were trained and empowered to rapidly initiate HAART on HIV-infected infants. In facilities where HAART was not available, ART was integrated into ANC/MCH clinics, or referrals to HAART sites were facilitated.

### 2.6. Setting

 FACES delivers a comprehensive HIV prevention, care and treatment program in Nyanza Province, where HIV prevalence and infant mortality are highest in the country at 14.9% and 95 per 1000, respectively [21, 22]. FACES, a collaboration between the University of California San Francisco (UCSF), the Kenya Medical Research Institute (KEMRI), and the Kenyan MOH, works to build the capacity of the Kenyan government to implement quality HIV services through targeted technical support, training, and health care workforce support.

This intervention was implemented within 119 clinics in 5 districts in Nyanza Province. Clinics were included if they were supported by FACES and currently implementing PMTCT services. Eighty-two (69%) of the 119 clinics were also providing HAART at the time of RRI implementation. All levels of facilities were included including 6 district hospitals, 5 subdistrict hospitals, 26 health centers, and 82 dispensaries.

### 2.7. Measurement

 Site-level data were captured at baseline, covering 12 weeks (October 2010–January 2011) and compared to the RRI 12-week period (April 2011–June 2011), and to a 12-week post-RRI period (July 2011–September 2011) to examine changes in testing and uptake of services across the sites. December 2010 data were omitted from baseline due to the shortened work month. Data were collected using routine program PMTCT monthly data collection tools. As part of standard care, maternal-child health (MCH) staff documented daily patient care in ANC, maternal (MAT), postnatal (POST), and HIV-exposed infant (HEI) MOH registers. PMTCT variables were extracted from the registers and entered in aggregated form into PMTCT monthly data collection tools. Key outcomes were assessed at baseline and were compared to RRI and post-RRI periods including (1) number of pregnant women counseled and HIV tested in ANC, (2) proportion of women tested for HIV in ANC who had a male partner HIV tested in ANC, (3) proportion of women tested in ANC with confirmed HIV-positive results, (4) proportion of HIV-positive women in ANC who had blood taken for CD4 testing in ANC, (5) proportion of HIV-positive women who initiated on HAART in ANC, (6) the number of exposed infants that had a HIV PCR test as a proportion of the number of HIV-positive women in ANC, (7) proportion of exposed infants that were HIV-positive, and (8) proportion of HIV-positive infants initiated on HAART.

### 2.8. Statistical Methods

 Data obtained during the baseline, RRI and post-RRI periods were compared to assess whether there were significant changes during the three periods using pre-post cohort analysis using Stata 10 (StataCorp, College Station, TX, USA). Temporal changes in indicators were considered significant at a *P* value of <0.05. The risks, risk difference, and risk ratios (95% confidence Intervals) were reported for each indicator with the RRI baseline period as the reference point.

### 2.9. Ethical Review

 The FACES' program evaluation protocol was reviewed and approved by the KEMRI Ethical Review Committee, UCSF Committee on Human Research, and Centers for Disease Control and Prevention NCHHSTP ADS/ADLS Review Committee.

## 3. Results

### 3.1. HIV Counseling and Testing

During the baseline period, 8591 women were newly HIV-tested in the ANC. During the sixty-day RRI, 9123 women were counseled and tested, with a significant increase of 9.1% (95% CI 8.8%–9.8%). In the post-RRI period, women counseled and tested dropped to 8068, below baseline levels, with a 6.1% decrease (95% CI 5.6%-6.6%). The proportion of women testing HIV-positive varied slightly from baseline (19.3%) during the RRI (20.7%) and post-RRI (18.9%) periods (RR 1.07, 95% CI 1.01–1.14; RR 0.98, 95% CI 0.92–1.04, resp.).

### 3.2. HAART Eligibility Assessment and HAART Uptake

HAART eligibility assessment was measured as the number of HIV-positive women receiving CD4 test results. CD4 testing increased from a baseline of 980 (59.0%) CD4 tests out of 1662 HIV-positive pregnant women to 1258 (66.6%) tests out of 1890 women during the RRI phase (RR 1.13, 95% CI 1.1-1.2) and remained higher in the post-RRI period; 966 (63.3%) CD4 tests were performed out of 1526 HIV-infected women (RR 1.1, 95% CI 1.0-1.1) (Table 1). The relative proportion of HAART uptake compared to all HIV-positive women improved significantly by 40% from 13.7% at baseline to 19.7% (RR 1.4, 95% CI 1.2–1.7) during the RRI period and by 60% to 21.7% (RR 1.6, 95% CI 1.4–1.8) in the post-RRI period (Table 1).

### 3.3. Early Infant Diagnosis and HAART Uptake in HIV-Positive Infants

During the baseline period, 768 (46%) HIV-PCR tests from HIV-exposed infants were performed amongst 1662 HIV-positive pregnant women, which significantly increased to 1149 (61%) PCR tests out of 1890-HIV positive pregnant women (RR 1.3, 95% CI 1.2–1.4) during the RRI and to 1327 (87%) out of 1526 positive women (RR 1.9, 95% CI 1.8–2.0) in the post-RRI period. This demonstrates a significant increase in EID uptake; with HIV exposed infants 30% more likely to be tested during RRI and 90% more likely to be tested post-RRI. HIV-positive results by PCR varied slightly between 12.1%, 13.8%, and 11.5% during baseline, RRI, and post-RRI periods, respectively. The proportion of eligible infants initiated on HAART went from 54.8% to 60.1% (RR 1.1, 95% CI 0.9–1.4) during the RRI and to 69.0% post-RRI (RR 1.3, 95% CI 1.0–1.6) reflecting that infected infants were 30% more likely to initiate HAART post-RRI (Table 1).

### 3.4. Male Partner Involvement

Male partners HIV testing in the ANC improved more than two-fold from 7.7% of all women counseled and tested at baseline to 16.4% during the RRI (RR 2.1, 95% CI 2.0–2.3). In the post-RRI period, the proportion of men tested for HIV decreased to 11.5% but remained 1.5 times above baseline figures (RR 1.5, 95% CI 1.4–1.7) (Table 1).

## 4. Discussion

This RRI was implemented to address key PMTCT service challenges and to specifically increase uptake of HAART in HIV-positive pregnant women and infants. The RRI was associated with a 13% increase in assessment for HAART eligibility via improved CD4 testing, which translated to 66.6% of HIV positive pregnant women getting CD4 testing. CD4 testing uptake was sustained in the post-RRI period. More encouraging was uptake of HAART for pregnant women, which increased during the RRI by 44% from baseline and by 58% post-RRI, showing an increased capacity for HAART uptake. By improving HAART uptake, facilities are closer to reaching the 30% of expected eligible HIV-positive women. Improved clinical staging likely also contributed to increased HAART uptake however, government data collection tools used during the intervention did not document clinical staging making it difficult to determine its contribution. Due to the cohort nature of this evaluation, it is not possible to determine the eligibility of each individual woman; however it is clear that despite substantial progress in HAART initiation, many are still not accessing this critical service since uptake still lags below 30%. Reasons likely include incomplete assessment for eligibility, failure to return to the ANC clinic for CD4 results and HAART initiation (despite tracing efforts and counseling), and lack of availability of HAART services at smaller health facilities resulting in unsuccessful linkage to a health facility providing full HIV services including HAART.

Early infant diagnosis via PCR testing is a key step in increasing uptake of HAART for HIV-infected infants. The RRI was associated with a 30% increase in PCR testing from baseline, which further improved by 90% from baseline compared in the post-RRI period. Additionally, review of many facility records showed a faster turnaround time for PCR testing (data not shown). Along with improved identification of HAART-eligible infants, actual HAART uptake increased modestly 1.1-fold during the RRI but 1.3-fold in the post-RRI period. Similar to HAART uptake amongst HIV-positive pregnant women, uptake of HAART in HIV-positive infants remained a challenge despite RRI efforts. Further evaluation of barriers to HAART uptake should be reviewed and best practices implemented.

Male involvement as measured by partner HIV testing also significantly increased during the intervention. Male partner HIV testing increased 2.1-fold during the RRI and was sustained at 50% increased level of testing in the post-RRI period. However, the absolute percentage of women attending ANC whose partner came for HIV testing remained low (<20%). We believe this is an essential component to PMTCT service uptake and retention. Women who fail to disclose to their partners due to fear, stigma, or denial are much more likely to default care, and less likely to deliver in a health facility [14, 15]. HIV couples counseling and testing provide a facilitated environment for HIV testing where issues of blame, discordance, and future care options are explained [14, 15].

We believe community mobilization as well as leadership and involvement of the MOH, though difficult to measure, contributed significantly to success of the RRI. Ultimately, health-seeking behavior is determined at an individual level but heavily influenced by family and community attitudes and behaviors. The use of opinion leaders such as chiefs, district administration, peer educators, and community health workers likely sensitized the community to the importance of PMTCT services. Which particular activities within community mobilization are most effective in changing attitudes, and behavior is a key research topic for future evaluations. Given the setting of the RRI primarily within government health facilities in rural communities and the need to build sustainable and lasting interventions, it was essential to have MOH ownership and leadership for this intervention.

Strengths of this study include its applicability and reproducibility in other PMTCT programs. Governments, nongovernmental organizations (NGOs), and other partners implementing PMTCT services can adapt the RRI concept to their particular settings and assess its impact. Weaknesses include difficulty in assessing the impact of particular strategies including community mobilization and male partner involvement. Additionally, this was a nonrandomized intervention. Community and facility level confounders cannot be ruled out. Furthermore, data were collected by facilities and technical staff supporting those facilities that had an interest in seeing improvements based on their work. Routine data quality audits were conducted at a portion of sites by an independent team, the monitoring and evaluation officers.

The ultimate goal of PMTCT programs is to provide high quality, cost-effective services that translate into saved lives and reduced morbidity among women and children. While the RRI concept focuses on intensive intervention over a short period, the program was designed to use strategies that build healthcare worker capacity and strengthen overall systems such as laboratory networking that lead to sustained improvements in outcomes. While we found that the RRI was associated with a relatively short-term sustained improvement in most indicators, further research is required to determine its longer term impact, the need for repeat RRIs, and newer evidence-based practices such as ANC and HIV care and treatment service integration. The followup phase of the RRI is ongoing and includes routine support of implementation of PMTCT services, which includes support supervision, mentorship, technical support, and CQI activities. A truly integrated multidisciplinary approach which engages key stakeholders including the local community, such that promoted during the RRI, will play an important role towards reaching the goal of eliminating mother-to-child transmission of HIV and improving health outcomes for HIV-positive women.

## Figures and Tables

**Table 1:** Comparison of key PMTCT outcomes at baseline, and during the Rapid Results Initiative (RRI) and the post-RRI periods.

	Baseline period	RRI period		Post-RRI period	
	Oct 2010–Jan 2011*	Apr–Jun 2011		Jul–Sep 2011	
	*N* (%)	*N* (%)	Risk ratio (95% CI)^+^	*N* (%)	Risk ratio (95% CI)^+^
* Maternal outcomes*					
HIV testing	8591	9123		8068	
HIVpositive	1662	1890		1526	
CD4 testing	980/1662 (59.0%)	1258/1890 (66.6%)	1.1 (1.1-1.2)^*∧*^	966/1526 (63.3%)	1.1 (1.0–1.1)
HAART initiation	228/1662 (13.7%)	373/1890 (19.7%)	1.4 (1.2–1.7)^*∧*^	331/1526 (21.7%)	1.58 (1.4–1.8)^*∧*^
*HIV-exposed infant outcomes*					
PCR testing	768/1662 (46.2%)	1149/1890 (60.8%)	1.3 (1.2–1.4)^*∧*^	1327/1526 (87.0%)	1.9 (1.8–2.0)^*∧*^
PCR positive	93/768 (12.1%)	158/1149 (13.8%)	1.1 (0.9–1.4)	152/1327 (11.5%)	0.9 (0.7–1.2)
HAART initiation	51/93 (54.8%)	95/158 (60.1%)	1.1 (0.9–1.4)	105/152 (69.0%)	1.3 (1.0–1.6)
*Male partner engagement outcome*					
HIV testing	660/8591 (7.7%)	1496/9123 (16.3%)	2.1 (2.0–2.3)^*∧*^	939/8068 (11.6%)	1.5 (1.4–1.7)^*∧*^

*December 2010 data excluded.

^+^Results compared to baseline survey period.

^*∧*^Statistically significant.
